# AMONDYS 45 (Casimersen), a Novel Antisense Phosphorodiamidate Morpholino Oligomer: Clinical Considerations for Treatment in Duchenne Muscular Dystrophy

**DOI:** 10.7759/cureus.51237

**Published:** 2023-12-28

**Authors:** Megan E Vasterling, Rebecca J Maitski, Brice A Davis, Julie E Barnes, Rucha A Kelkar, Rachel J Klapper, Hirni Patel, Shahab Ahmadzadeh, Sahar Shekoohi, Alan D Kaye, Giustino Varrassi

**Affiliations:** 1 School of Medicine, Louisiana State University Health Sciences Center, New Orleans, USA; 2 School of Medicine, Medical University of South Carolina, Charleston, USA; 3 Radiology, Louisiana State University Health Sciences Center, Shreveport, USA; 4 Anesthesiology, Louisiana State University Health Sciences Center, Shreveport, USA; 5 Pain Medicine, Paolo Procacci Foundation, Rome, ITA

**Keywords:** gene therapy, exon skipping, duchenne muscular dystrophy, casimersen, amondys 45

## Abstract

AMONDYS 45 (casimersen) is an antisense oligonucleotide therapy used to treat Duchenne muscular dystrophy (DMD), a rare genetic disorder characterized by a mutation in the DMD gene. Symptoms include progressive muscle weakness, respiratory and cardiac complications, and premature death. Casimersen targets a specific mutation in the DMD gene that results in the absence of dystrophin protein, a key structural component of muscle fibers. While there is currently no cure for DMD, exon-skipping therapy works by restoring the reading frame of the mutated gene, allowing the production of a partially functional dystrophin protein. Clinical trials of casimersen have shown promising results in increasing dystrophin production, as measured by polymerase chain reaction (PCR) droplets when compared to placebo. In a randomized double-blind trial, patients who received casimersen had significantly higher dystrophin levels when compared to those who received placebo. Casimersen therapy is administered through repeated intravenous infusions, although the optimal dosage and duration of treatment are still under investigation. Based on the completed and ongoing clinical trials, casimersen has been well tolerated, with most adverse events being mild and unrelated to casimersen. In 2021, AMONDYS 45 (casimersen) received approval from the US Food and Drug Administration (FDA) for the treatment of Duchene muscular dystrophy in patients with a mutation of the DMD gene that is amenable to exon 45 skipping. These collective findings indicate that casimersen has the potential to elicit functional changes in individuals with DMD, although further studies are necessary to comprehensively evaluate the specific functional improvements. Regardless, the FDA approval and ongoing clinic trials mark a significant milestone in the development of DMD treatments and offer hope for those affected by this debilitating disease.

## Introduction and background

Duchenne muscular dystrophy (DMD) is a genetic disorder that affects approximately one in 5,000 boys aged five to nine in the United States [1]. It is caused by a mutation in the DMD gene, which codes for dystrophin, an essential structural protein of muscle fibers. Without functional dystrophin, muscle fibers become damaged and eventually die, leading to progressive muscle weakness and wasting [2]. Most children are diagnosed with DMD by age five, as symptoms typically begin in early childhood. Initial signs may include delayed motor milestones, such as difficulty walking or running, and frequent falls. As the disease progresses, children may develop difficulty climbing stairs, standing up, and lifting objects. By their teens, most children with DMD require the use of a wheelchair; by early adulthood, the disease can be life-threatening, as it can affect the muscles involved in breathing and heart function [1,2]. While there is currently no cure for DMD, available treatments mitigate symptoms and slow disease progression. One such treatment, AMONDYS 45 (casimersen), was confirmed by the US Food and Drug Administration (FDA) in 2021 [3]. Casimersen works through a type of genetic therapy known as exon skipping, which targets specific mutations in the DMD gene to restore the production of functional dystrophin protein. Casimersen is indicated in patients with a confirmed DMD gene mutation that is amendable to exon 45 skipping, allowing the production of a shortened, yet functional, dystrophin protein. It is important to note that casimersen is not a cure for DMD, nor does it address the underlying genetic mutation. Instead, it aims to slow the disease's progression and improve patients' quality of life. Clinical trials have found that patients who received casimersen demonstrated significant increases in dystrophin production compared to placebo [4]. In the present investigation, therefore, we review the presentation, epidemiology, pathophysiology, and currently available treatments for DMD, with particular emphasis on emerging technologies, including AMONDYS 45 (casimersen).

## Review

Duchenne muscular dystrophy presentation, epidemiology, pathophysiology

DMD is a class of inherited disorders with abnormal muscle function as a result of mutations in the DMD gene [5]. The most common mutations are frameshift or nonsense mutations, leading to premature truncation of the dystrophin protein [6]. Dystrophin is present in striated muscle, cardiac muscle, the brain, and the retina [2], where it provides myofiber structural stability [5]. While this protein usually serves to protect muscle from mechanical injury, muscle fibers in patients with the DMD mutation degenerate and undergo a cyclical process of necrosis and regeneration, ultimately leading to muscle weakness [2]. With their increased membrane fragility, these muscles are more susceptible to oxidative damage and dysregulation of calcium homeostasis.

DMD disorders present in an X-linked recessive manner, most commonly presenting in males. It is estimated that one in 3600 males is born with DMD each year [2]. Symptoms present in approximately five stages, beginning within the first few years of life [5]. The pre-symptomatic phase begins with a rise in creatine kinase, along with a family history of muscular dystrophies. The early ambulatory phase will present in young childhood with proximal musculature weakness, predominantly in the pelvic girdle. There will also be a positive Gowers sign, in which the child will use their arms to push off their knees to stand.

Additionally, pseudohypertrophy of calf muscles may be present, in which muscle fibers are replaced by adipose tissue. In the late ambulatory phase, the child has problems walking independently and may start to see a decline in their forced vital capacity, ultimately causing hypoxemia. In the early non-ambulatory phase, the child will be able to maintain their posture, but signs of scoliosis may appear. Finally, the late non-ambulatory phase presents with children being dependent on a wheelchair around ages 10-12. Respiratory and cardiac failure will also present around this age [5].

Overall, DMD patients are at an increased risk of fractures, heart failure, nutritional deficiencies, and pharyngeal weakness. It is very uncommon for urinary or bowel incontinence to be present [2]. The long-term consequences of this disease are due to functional ischemia, free radical damage, and calcium overload [6]. The damaged tissue is unable to withstand such damage and cannot properly repair itself. The life expectancy of these patients can range from 20 to 40 years of age [6].

Current management of Duchenne muscular dystrophy

While there is no cure for DMD at this time, supportive treatments can help improve the quality of life and potentially prolong lifespans [5]. Given its impact on numerous organ systems, a multidisciplinary DMD treatment approach is optimal. The current standard of care includes corticosteroids for controlling both inflammation and the subsequent necrosis of muscle fibers. Patients on corticosteroids have been shown to prolong independent ambulation while maintaining muscle strength for longer periods. They have also been found to reduce incidences of severe scoliosis and the subsequent need for surgical interventions [6]. However, there is some debate on the side effects versus the benefits associated with long-term corticosteroid use in children. Corticosteroids may predispose patients to compression fractures, thereby worsening their symptoms [6]. Oxandrolone, an anabolic steroid, was approved in 2006 and found to antagonize glucocorticoid receptors but is not widely used today [5]. Additional assessments also include periodic respiratory function tests due to DMD-induced pharyngeal and respiratory muscle weakness [6]. Patients may require assisted coughing and mechanical ventilation to assist with hypoventilation. Since dilated cardiomyopathy is a common problem associated with DMD, angiotensin-converting enzyme (ACE) inhibitors are also recommended for those with ventricular dysfunction. The reduction in afterload can reduce symptoms and reduce fibrosis associated with angiotensin II-associated cardiac remodeling. Orthopedic DMD management includes physical therapy and physiotherapy to prevent contractions, especially of the Achilles tendon, knee, and hip flexors [6].

While the above therapies may provide symptomatic management for the lack of dystrophin, new therapies such as read-through therapy and exon skipping revolve around restoring dystrophin expression and function [7].

Read-through therapy may be used in patients with nonsense mutations, which are mutations that cause an early stop codon and a non-functional dystrophin protein. Moreover, the mRNA that is generated despite the nonsense mutation is destabilized by nonsense-mediated mRNA decay (NMD). Inactivating any factor related to this pathway would seem to be beneficial in stabilizing the mRNA transcript, and this is the primary concept behind read-through therapies. Studies have shown that aminoglycosides, specifically gentamycin, can restore dystrophin expression in patients with these nonsense mutations [7]. Ataluren, a therapeutic agent that promotes the suppression of nonsense mutations, has been shown to allow the production of full-length functional proteins [8]. It has also been shown to improve contractile function and dystrophin expression in animal models of DMD [9]. Additionally, clinical trial results have demonstrated that ataluren significantly delays the age at loss of ambulation and worsens performance in timed function tests in patients with DMD [10].

Exon skipping is used in patients with frameshift mutations. In this model, antisense oligonucleotides (ASOs) skip targeted exons to correct reading frames and restore dystrophin stores. While this does lead to a shorter dystrophin protein, the phenotype presents with less severe symptoms than the complete absence of dystrophin.

Lastly, the transplantation of cells capable of producing functional dystrophin proteins, such as satellite cells and myoblasts, holds promise for the treatment of DMD [11,12]. However, the lack of animal models for conducting large-scale preclinical studies using human cells, including biochemical and functional tests, hinders research progress in this area [13]. Moreover, the defective proliferative capacity of satellite cells, which are precursors of mature muscle fibers, has been observed in DMD patients, further complicating the development and assessment of cell-based therapies [14].

AMONDYS 45 (casimersen) clinical pharmacology

AMONDYS 45 (cashmere) is specifically indicated for DMD patients with a confirmed genetic mutation amenable to exon 45. The recommended dose of Casimersen is 30 mg/kg/week, administered intravenously over approximately 30 minutes to an hour [3].

Mechanism of action

Casimersen is an antisense phosphorodiamidate morpholino oligomer (PMO) drug designed to target genetic mutations that are amenable to exon 45 skipping in patients with Duchenne muscular dystrophy (DMD) [3]. Casimersen is theorized to enter the dystrophic muscle cells via pores in their leaky membranes, with a higher affinity for entering immature fibers. Once inside dystrophic muscle cells, casimersen enters the nucleus and binds to the DMD pre-mRNA 3′ splice site. As a result, it blocks the splicing signal near exon 45, causing the spliceosome to skip it. By excluding exon 45 during mRNA processing and restoring the frame, a dystrophin isoform protein is produced that is similar to the protein found in Becker's muscular dystrophy [15]. The new dystrophin isoform could potentially alter the severity of disease in DMD patients and allow for symptoms more similar to Becker’s muscular dystrophy [16].

Pharmacodynamics

In the ongoing phase 3 ESSENCE trial, muscle biopsies taken at 48 weeks showed that casimersen-treated patients showed significant increases in exon 45 skipping compared to placebo-treated patients and baseline, as assessed by droplet digital polymerase chain reaction (PCR). Casimersen-treated patients also showed similar mean dystrophin levels compared to placebo-treated patients and baseline via western blot analysis. It was found that the restored dystrophin protein was correctly localized to the sarcolemma in casimersen-treated patients. The increase in dystrophin positively correlated with exon skipping, linking the new dystrophin production to exon skipping [17].

Pharmacokinetics

The pharmacokinetics of casimersen were evaluated in DMD patients using intravenous doses ranging from 4 mg/kg/week to the recommended 30 mg/kg/week. Results demonstrated that casimersen’s maximum concentration (Cmax) was observed at the end of infusion after a single IV dose, with exposure increasing proportionally to the dose. There was no plasma accumulation with once-weekly dosing. Inter-subject variability for Cmax and area under the curve (AUC) ranged from 12% to 34% and 16% to 34%, respectively. The drug was found to bind to human plasma proteins non-concentration-dependently, ranging from 8.4% to 31.6%. After administering a 30 mg/kg dose intravenously, the mean apparent volume of distribution at steady state (Vss) was 367 mL/kg (%CV = 28.9), and the plasma clearance (CL) of casimersen was 180 mL/hr/kg. The elimination half-life (t1/2) was 3.5 hours (SD 0.4 hours). Casimersen is metabolically stable in human hepatic microsomal incubations, with no metabolites detected in plasma or urine. The drug is mostly excreted unchanged in the urine, with more than 90% of the drug excreted in urine and negligible fecal excretion in a clinical study with radiolabeled casimersen [3].

Adverse Effects

Due to its low accumulation in the plasma and short half-life, casimersen is unlikely to have any drug interactions or major effects on enzymes and transporters. One study found that upper respiratory tract infections, cough, pyrexia, headache, arthralgia, and oropharyngeal pain events occurred more frequently in patients who received casimersen versus placebo. Casimersen has no contraindications; however, patients with decreased kidney function should be monitored closely [3].

Clinical Studies

A multicenter randomized, double-blinded, placebo-controlled dose-titration trial was done to evaluate the safety and tolerability of casimersen, with the secondary objective of characterizing the plasma pharmacokinetics of the drug. Twelve participants aged 7-21 who had limited ambulation or were non-ambulatory were enrolled in the study, with 11 participants completing the trial. Participants were randomized 2:1 to weekly casimersen infusions at escalating doses of 4, 10, 20, and 30 mg/kg or placebo. Administration of casimersen or placebo consisted of a 12-week, double-blinded dose titration period, followed by an open-label extension for up to 132 weeks. Safety assessments included adverse events, vital signs, physical examinations, clinical laboratory evaluations, electrocardiograms, and echocardiograms. The severity of all adverse events was assessed as mild, moderate, or severe, and it was determined if they were related or unrelated to the study. Adverse events were considered treatment-emergent if they started, worsened, or became serious either on or after the start of the first infusions and within 28 days after the last dose of the study drug, or before receiving the first dose in the extension study [18]. In more than 20% of patients, adverse drug reactions included upper respiratory tract infections, headache, cough, arthralgia, pyrexia, or oropharyngeal pain. Less common adverse drug reactions were nausea, ear pain and/or infection, dizziness, light-headedness, and post-traumatic pain [19]. All participants had at least one treatment-emergent adverse event (TEAE), and TEAEs had the same reported frequency for casimersen and placebo. There was no indication of more adverse events occurring at higher doses, and no participants reduced their dosage or discontinued the study due to TEAEs. Overall, casimersen 30 mg/kg was determined to have an acceptable safety profile and was well tolerated in patients with Duchenne's muscular dystrophy with confirmed mutations due to exon 45 skipping. A non-randomized extension study to assess the safety and tolerability of long-term therapy with casimersen or golodirsen in DMD patients is currently in Phase 3 and has 260 participants aged seven to 23 years [20]. Patients that have completed a clinical trial with casimersen will receive an open-label casimersen 30 mg/kg IV infusion for up to 144 weeks. The primary outcome measure is the number of patients with serious adverse events.

An ongoing Phase 3 double-blinded, placebo-controlled, multi-center study evaluates the efficacy and safety of casimersen or golodirsen over 96 weeks, followed by a 48-week open-label period. There were 229 eligible patients aged seven to 13 years old with mutations attributable to exon 45 skipping were randomized 2:1 to casimersen 30 mg/kg or placebo once weekly [21]. All participants will undergo a muscle biopsy for baseline measurement and a second muscle biopsy at week 48 or week 96. Clinical efficacy will be investigated at regularly scheduled study visits. Efficacy measures will include functional tests, such as the six-minute walk test [21]. Safety will be evaluated through a collection of laboratory tests, adverse events, vital signs, physical examinations, electrocardiograms, and echocardiograms throughout the study. Exon 45 skipping will be assessed by droplet digital PCR. The anticipated primary study outcome is a change from baseline in the total distance walking during the six-minute walk test at week 96. Other changes from baseline are the anticipated secondary outcomes, such as total distance walking during the six-minute walk test at week 144, dystrophin levels, forced vital capacity percent, time to loss of ambulation, and the participant’s ability to rise independently from the floor. Thus far, muscle biopsy with subsequent western blot analysis has shown significant increases in mean dystrophin levels after 48 weeks of casimersen treatment. Increased dystrophin showed a positive correlation with exon skipping. Immunofluorescence staining suggested that casimersen-treated patients have correct sarcolemmal localization of the restored dystrophin [17]. Casimersen has been well tolerated, with most adverse events being mild and unrelated to casimersen. The safety and efficacy of casimersen are continuing to be evaluated, and the suspected completion of the study is in October 2025 [21].

A 48-week open-label study in Phase 2 is ongoing to investigate the efficacy and safety of treatment with casimersen, eteplirsen, and golodirsen in DMD patients carrying eligible exon duplication of exons 45, 51, or 53, respectively [22]. Three participants over the age of six months old are currently enrolled. The primary outcome measures include the change in dystrophin expression from baseline following casimersen treatment and the development of unacceptable toxicity. Changes in dystrophin expression will be assessed by western blot quantification of protein in muscle tissue from biopsy. Documentation of adverse events will be used as a measurement of unacceptable toxicity development. The study is estimated to be complete in September 2023 (Table 1).

**Table 1:** AMONDYS 45 (casimersen) clinical trials

Author (Year)	Groups Studied and Intervention	Results and Findings	Conclusions
Study 1: Wagner et al. (2021) [18]	Patients with limited ambulation received escalating doses of 4, 10, 20 and 30 mg/kg casimersen weekly vs placebo.	Adverse events occurred in all casimersen- and placebo-treated patients, although there were no serious adverse events attributed to casimersen. The drug plasma concentration increased with an increasing dose. Plasma concentrations declined similarly over the 24 hours following the infusion.	Casimersen 30 mg/kg was determined to have an acceptable safety profile and was well tolerated.
Study 2: Iannaccone et al. (2022) [17]	Patients with exon 45 skip-amenable mutations were randomized 2:1 to take casimersen 30 mg/kg or placebo IV once weekly.	Patients treated with casimersen had increased exon 45 skipping on PCR. Additionally, there were increased levels of dystrophin on western blot analysis at 48 weeks when compared to placebo.	Casimersen significantly increases exon 45 skipping resulting in increased dystrophin production.
Study 3: Sarepta Therapeutics, Inc (2022) [20]	Casimersen or golodirsen was administered at 30 mg/kg weekly in patients with DMD amendable to exon 45 and 53 skipping, respectively.	Ongoing – results are not yet available.	
Study 4: Flanigan K. (2022) [22]	Patients with Duchenne muscular dystrophy and known exon 45, 51, or 53 duplications were treated with casimersen, eteplirsen, or golodirsen, respectively	Ongoing – results are not yet available.	

Discussion

DMD is a rare genetic condition with an estimated prevalence of one in 3600 male births worldwide [2,23-29]. An absence or deficiency of dystrophin protein leads to progressive muscle weakness, respiratory and cardiac complications, and premature death [2,5]. Despite advances in treatment, there is currently no cure for DMD, and available treatments only aim to slow disease progression and manage symptoms. Currently, available treatment options for DMD include glucocorticoids, exon-skipping therapies, and gene therapy. Glucocorticoids have been shown to prolong the ability to walk and delay the onset of respiratory and cardiac complications. Unfortunately, however, they pose significant risks with long-term use in children [6,30-34]. The treatment landscape for DMD has seen significant advancements with the development of exon-skipping therapies such as casimersen. These therapies aim to restore protein expression in neuromuscular conditions by targeting specific exon mutations associated with DMD [35-36].

Casimersen, an antisense oligonucleotide therapy that targets exon 45 of the DMD gene, restores the reading frame and allows the production of a partially functional dystrophin protein. In clinical trials, casimersen has demonstrated efficacy in increasing dystrophin production at 48 weeks as measured by droplet digital polymerase chain reaction (PCR)(n=27, P<0.001 and n=16, P=0.808 respectively). Similarly, western blot analysis (0.93% vs. 1.74% of normal; P<0.001) of muscle tissue confirms increased dystrophin production when compared with placebo (mean difference=0.59%; P=0.004) [17]. These findings collectively suggest that casimersen and other exon-skipping therapies have the potential to induce functional changes in DMD patients. However, further research and clinical trials are necessary to comprehensively evaluate the specific functional improvements associated with casimersen and its impact on the overall quality of life for individuals with DMD. Ultimately, the approval of casimersen by the FDA in 2021 represents a critical milestone in the treatment of DMD, offering hope to patients with specific exon mutations amenable to exon skipping. Despite the promising results of casimersen in clinical trials, there are still limitations to its use, for example, the need for repeated intravenous infusions and the possibility of adverse effects such as renal dysfunction [3]. While these gene-specific therapies have the potential to transform the management of DMD, further studies also warrant investigation into the optimal dosage, duration of treatment, and potential long-term side effects [18-20].

## Conclusions

In conclusion, while there is currently no cure for DMD, emerging technologies such as AMONDYS 45 (casimersen) offer hope for slowing the progression of the disease and improving the quality of life for those affected by DMD. This review provides a comprehensive exploration of the effects of accelerated approval of casimersen for the treatment of DMD. While this represents a significant advancement in the field of genetic therapies, few publications fully describe the current state of research regarding the specific targeting of exon 45 skipping by casimersen. This review serves as the first publication to not only summarize existing knowledge of molecular mechanisms, efficacy, and safety profiles but also provide an up-to-date examination of casimersen's clinical trial outcomes. By consolidating this information, this review serves as an invaluable resource for researchers, clinicians, and pharmaceutical professionals seeking a nuanced understanding of casimersen's current state of research and its potential future implications for patients with DMD.
